# Defining the Distinct Skin and Gut Microbiomes of the Northern Pike (*Esox lucius*)

**DOI:** 10.3389/fmicb.2019.02118

**Published:** 2019-09-12

**Authors:** Elizabeth M. Reinhart, Benjamin J. Korry, Aislinn D. Rowan-Nash, Peter Belenky

**Affiliations:** Department of Molecular Microbiology and Immunology, Division of Biology and Medicine, Brown University, Providence, RI, United States

**Keywords:** fish, microbiota, *Cetobacterium*, 16S rRNA, aquatic, carnivore, communities, next-generation sequencing

## Abstract

The microbiome of freshwater fish has important implications for both commercial and recreational fishing because it can have significant impacts on host heath, spoilage rates, and susceptibility to disease. The aqueous environment serves as a possible avenue for continuous introduction of microbes to an animal host, but little is known about how the surrounding microbiota contribute to piscine microbiomes. To better understand the composition of the fish microbiome exposed to the natural environment, we profiled the microbial composition of the gut and the skin mucosal surface (SMS) of northern pike (*Esox lucius*) and the surrounding river water. We collected fish samples from eight sites along a single river in southwestern Quebec, Canada and analyzed the microbial composition via 16S rRNA sequencing. Our results reveal robust taxonomic differences between the SMS and the gut, indicating a divergence between the microbiomes. The gut community was characterized by a lower alpha diversity compared to the SMS and a large proportion of *Cetobacterium*, a genus previously linked to carnivorous species. On the other hand, the SMS was more similar to the water than the gut at the family level but divergent at lower taxonomic levels, with fewer than 30% of amplicon sequence variants (ASVs) shared between the SMS and water. In total, our results suggest the establishment of distinct communities across the two fish sites, as well as a clear separation from the microbes in surrounding waters. These data indicate that despite continuous exposure to water, pike are able to establish and maintain unique microbial communities.

## Introduction

Understanding the bacterial composition of fish microbiota is important for commercial, and recreational fisheries because it is known to have significant impacts on host health, spoilage rates, and susceptibility to disease (Gram and Huss, 1996; Gomez and Balcazar, 2008; Llewellyn et al., 2014; Piazzon et al., 2017; Odeyemi et al., 2018). By comparing these communities to other freshwater microbiomes, we can deepen our perspective on how these communities establish and are maintained in disparate organisms. Interestingly, many of the concepts developed in terrestrial microbiomes also hold true in piscine communities (Sullam et al., 2012). For example, as with mammals, gut communities are similar between fish at the same trophic levels and with similar diets (Muegge et al., 2011; Delsuc et al., 2014; Liu et al., 2016; Wang et al., 2018). While microbial composition varies among fish species, the most abundant phyla found in the gut microbiota of freshwater fish are typically Proteobacteria, Actinobacteria, Bacteroidetes, Firmicutes, and Fusobacteria (Desai et al., 2012; Nielsen et al., 2017; Burgos et al., 2018; de Bruijn et al., 2018; Wang et al., 2018). However, the community composition can differ dramatically between carnivorous, omnivorous, and herbivorous fish (Givens et al., 2015; Miyake et al., 2015). Overall, studies show that piscine gut microbial diversity tends to decrease from herbivores to omnivores, with the lowest diversity in carnivores (Wang et al., 2018).

Microbial communities on the piscine skin are also important for fish health, although they are less well-studied than the gut. The skin is coated in a viscous mucus rich in nutrients, and the microbes in this niche (the skin mucosal surface, or SMS) are key to a healthy mucosal barrier and thereby a stable immune system (Carda-Dieguez et al., 2017; Legrand et al., 2017; Reverter et al., 2018). SMS microbial communities are distinct from those of the gastrointestinal tract (Sylvain et al., 2016; de Bruijn et al., 2018) and while they are species-specific (Larsen et al., 2013), they tend to be dominated by Proteobacteria followed by lower levels of Bacteroidetes, Actinobacteria, Firmicutes, and Verrucomicrobia (Merrifield and Rodiles, 2015; Mohammed and Arias, 2015; Tarnecki et al., 2017). Initially, the SMS is seeded by bacteria in the water, but over time, the SMS community establishes an increasingly divergent microbiome (Uren Webster et al., 2019). Additionally, a number of environmental factors have been shown to shift the composition of the SMS, including salinity (Lokesh and Kiron, 2016; Carda-Dieguez et al., 2017), seasonality (Larsen et al., 2015; Ray, 2016), sediment (Hess et al., 2015), stress (Boutin et al., 2013), and pH (Sylvain et al., 2016).

The aquatic environment is thought to provide a crucial avenue for colonization, leading to the acquisition of environmental bacteria in both the gut and SMS microbial communities (Ingerslev et al., 2014; Galbraith et al., 2018). However, despite the introduction of bacteria from the surrounding waters, studies indicate that the piscine gut microbiome harbors a taxonomic composition that is unique from that of the environment (Semova et al., 2012; Sullam et al., 2015). For example, a 2013 study by Xing et al. (2013) found that the gut of the turbot (*Scophthalmus maximus*) shared just 29.45% of its operational taxonomic units (OTUs) with the surrounding water. The separation from the surrounding waters continues for microbiota on the SMS. A study conducted by Chiarello et al. (2018) revealed that across 44 species of reef fish, only 10% of OTUs found in SMS communities were also found in the surrounding water. Since the microbes differ between freshwater and saltwater, influenced by the abundance of salt (Sunagawa et al., 2015), it is possible that the level of overlap may be different in freshwater. While the SMS communities of wild freshwater fish have not been extensively compared to those of their environments, a study did look at the SMS microbiome of the catadromous species (*Anguilla anguilla*) in its freshwater life-stage. The work found that the SMS community was distinct from the surrounding water, with Vibrio, Actinobacteria, and Gammaproteobacteria found at vastly different proportions between the two communities (Carda-Diéguez et al., 2014; Carda-Dieguez et al., 2017). Other reports have found microbial overlap between the SMS and water microbiomes for captive species. A study by Carlson et al. (2017) found that the SMS of captive western mosquitofish, *Gambusia affinis*, shares 76.9% of families with the water, representing 99.8% of the SMS abundance. Although it is possible this trend could hold true for wild populations, previous studies have shown that the microbiota of captive fish differs from their wild counterparts (Baldo et al., 2015). Moreover, the properties of each body of water could result in measurably different establishment and persistence of a wild SMS community.

In this study, we focus on the microbiome of the northern pike (*Esox lucius*), a large-bodied carnivorous fish inhabiting freshwater lakes and rivers of the northern hemisphere. Due to its large size and wide distribution, the northern pike is a popular and economically important game fish across North America and Eurasia (Forsman et al., 2015; Arlinghaus et al., 2017), but the bacterial composition of its microbiome has not previously been characterized with next-generation sequencing. In this study, we profile and compare the microbial communities of the pike SMS and gut, as well as of the surrounding freshwater environment. We find that despite exposure to the highly diverse microbiota of the surrounding water, the SMS and gut of this species harbor unique microbial communities that are similar to those of other carnivorous fish.

## Materials and Methods

### Collection

Northern pike microbiome samples were collected from fish harvested by licensed recreational fishermen who gave permission to collect microbial swabs from their catch. Samples were obtained at eight locations, ranging 55 km, along a single river in Southwestern Quebec, Canada (for full list of coordinates, refer to Supplementary Table S1). This fast-flowing river has no nearby permanent settlements or significant industrial activity other than limited logging around some surrounding tributaries. This isolation makes it a particularly good location for this study, because the samples are minimally impacted by human activity. Fish were sampled within a 7-day period in August 2018 to reduce temporal variation. These samples were only collected from fish that did not have contact with other fish after they were caught to minimize cross-contamination of the SMS. The total length of the fish was then measured (Supplementary Table S1) and a SMS and gut sample were collected from each, except for the last fish, from which only a gut sample was collected. SMS samples were collected by swabbing a 3 cm^2^ region posterior to the pectoral fin on both sides with flocked sterile swabs (Puritan Diagnostics, ME, United States; Cat: 25-3206-H). The gut microbiome samples were collected by inserting a fecal swab (Puritan Diagnostics, ME, United States; Cat: 25-3206-H) 5 cm past the anus and rotating 5 times. Both SMS and gut microbiome samples were stored individually in Zymo Research Bashing Bead 1.5 mL tubes containing ZymoBIOMICS Lysis Solution (Zymo Research, Irvine, CA, United States.; Cat: S6012-50, D4300-1-40). At different sites along the river, three water samples were collected midstream from the surface of rapidly moving water, in order to assess the microbiota of the freshwater environment. The water samples were stored in sterilized containers and transported back to the lab for DNA extraction. For each of the water samples, 1 liter of water was filtered through a 0.22 um filter, and DNA was extracted from a 5 cm^2^ piece of filter. In total, 8 gut, 7 SMS, and 3 water samples were collected.

### Bacterial 16S rRNA Amplicon Sequencing

DNA was extracted from gut, SMS, and water samples using the ZymoBIOMICS DNA Miniprep Kit, according to manufacturer instructions (Zymo Research, Irvine, CA, United States; Cat: D4300). PCR amplification targeted the V4 region, using the 515F forward primer with per-sample barcodes and the 806R reverse primer, according to the Earth Microbiome Project 16S Illumina Amplicon Protocol (Caporaso et al., 2010, 2012; Walters et al., 2016; Thompson et al., 2017). Amplification was carried out with Phusion High Fidelity polymerase (New England BioLabs, Ipswich, MA, United States) with the following PCR parameters: 98 C for 3 min, followed by 35 cycles of amplification (98 C for 45 s, 50 C for 60 s, and 72 C for 90 s), and a final elongation step at 72 C for 10 min. Equal amplicon concentrations were pooled and purified using the Machery-Nagel NucleoSpin Gel and PCR Clean-Up kit (Machery-Nagel, Düren, Germany; Cat: 740609). Samples were sent for quality control and sequencing to the Rhode Island Genomics and Sequencing Center at the University of Rhode Island (Kingston, RI, United States).

Amplicons were paired-end sequenced (2 × 300 bp) on an Illumina MiSeq platform using a 600-cycle kit with standard protocols.

### Sequencing Analysis

A total of 1,810,940 raw reads was obtained across all samples [for raw reads and unique amplicon sequence variants (ASVs) per sample, refer to Supplementary Table S2]. The raw paired-end FASTQ files were imported into QIIME2 (version 2018.8)^1^. Demultiplexing was performed using the demux plugin, while filtering, trimming, denoising, and merging was performed using the DADA2 plugin (Callahan et al., 2016). We chose not to rarefy the reads to avoid loss of useful data (McMurdie and Holmes, 2013). A phylogenetic tree was generated using the phylogeny plugin, and taxonomy was assigned to all ASVs using the feature-classifier plugin with a naïve Bayes classifier trained on the 515F/806R region of 16S rRNA gene sequences from the Silva (version 132) database of reference sequences clustered at 99% sequence similarity (Gurevich et al., 2013). Afterward, the feature table, rooted phylogenetic tree, and representative sequences artifacts were exported from QIIME2 for further analysis in R. Diversity metrics were calculated in R (version 3.5.1) using the vegan (version 2.5-3)(Dixon, 2003) and phyloseq (version 1.26.1) (McMurdie and Holmes, 2013) packages. Lastly, within QIIME2, the denoised sequences were used to predict microbial function through the PICRUSt2 plugin (version 2.0.3-b) (Langille et al., 2013; Douglas et al., 2019). All figures were generated with Prism (ver. 7.0a, GraphPad, La Jolla, CA, United States) using relative abundances averaged across the SMS, gut, and water communities.

To analyze beta diversity between sample sources, we performed a PERMANOVA via the adonis function within vegan (version 2.5-3). We used the Galaxy module Linear discriminant analysis Effect Size (LEfSe, *p*-values < 0.05) (Segata et al., 2011) to determine taxa specifically enriched in the SMS, gut, and water communities. The non-parametric Mann–Whitney Test was used to determine statistical significance in the alpha diversities of the microbial communities and the relative abundance of *Cetobacterium* across samples (^∗∗∗^<0.001, ^∗∗^<0.01, ^∗^<0.05).

## Results

### Alpha and Beta Diversity

**FIGURE 1:**
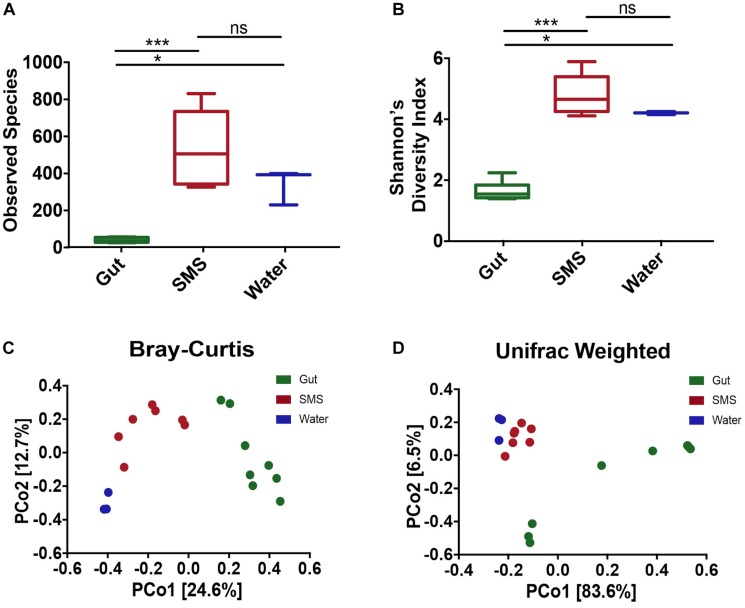
Alpha and Beta Diversity Analyses of the SMS, the Gut, and the Water Communities. Alpha-diversity was calculated using the metrics of **(A)** Observed ASVs and **(B)** Shannon’s Diversity Index. Statistical analysis was conducted on alpha diversities using Mann-Whitney tests. ns, not significant; *p* > 0.05, ^∗^*p* < 0.05, ^∗∗∗^*p* < 0.001. Beta-diversity was calculated and principle coordinate analysis (PCoA) was performed using the metrics of **(C)** Bray–Curtis Dissimilarity and **(D)** Weighted UniFrac. A PERMANOVA was used to detect significant differences in the beta-diversities. For the Bray–Curtis Dissimilarity PCoA, all communities clustered separately (*p*-value = 0.002, 0.007, 0.014 for SMS-gut, gut-water, and SMS-water, respectively). The weighted Unifrac revealed separate clustering between the SMS and gut as well as the gut and water communities (*p*-value = 0.001, 0.008), but the distance between the SMS and the water was non-significant (*p*-value = 0.103).

In this study, we used 16S rRNA sequencing to profile the microbiome found in the gut and on the SMS of the northern pike (*E. lucius*), as well as the microbiome of the surrounding water. When comparing diversity metrics, we found significant differences between the community makeup of the gut and the SMS microbiome. We first measured alpha diversity, or the diversity within the communities, using two metrics: observed ASVs, reflecting taxonomic richness, and the Shannon Diversity Index, incorporating both taxonomic richness and evenness (Figures 1A,B). For both alpha diversity metrics, the SMS community was significantly more diverse (*p*-value < 0.001) than the gut. The water also exhibited higher diversity than the gut (*p*-value < 0.05), but there were no significant differences in diversity between the water and SMS. These results indicate that both the SMS and the water harbor much more diverse communities than the gut microbiome.

To determine the variability within and between microbiome sources, we used multiple metrics. First, we utilized the Bray–Curtis dissimilarity index, which analyzes the relative abundances of the ASVs present. Second, we used the weighted Unifrac distance, which incorporates both phylogenetic relatedness and relative abundance. Principal coordinate analysis (PCoA) was used to plot both metrics. Across communities, the gut, SMS, and water microbiomes cluster separately with the greatest difference between the gut and water samples (Figures 1C,D; PERMANOVA values for Bray-Curtis and weighted Unifrac, respectively: SMS-gut *p*-value = 0.002, 0.001; Gut-water *p*-value = 0.007, 0.008). Water and SMS samples cluster relatively closely together based on the weighted Unifrac metric (*p*-value = 0.103). This may be consistent with the constant exposure of the skin mucosa to bacteria in the water; bacteria detected in SMS samples likely include taxa from the surrounding water. On the other hand, these samples cluster further apart based on the Bray–Curtis metric, suggesting the differences in communities may lie in closely related taxa. Neither fish length nor sample site had a clear impact on the variation between SMS and gut samples (Supplementary Figures S1C,D).

### Microbial Composition – Phyla

**FIGURE 2:**
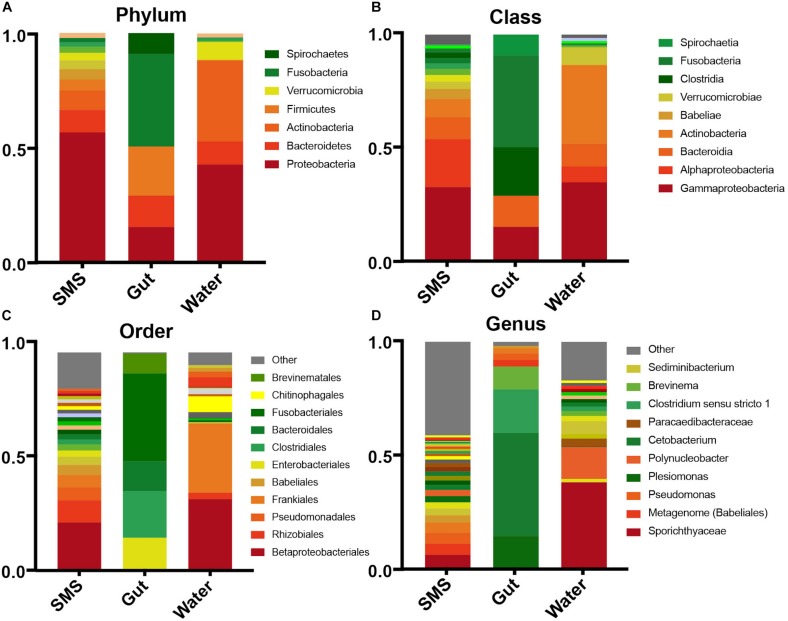
Taxonomic abundances within the SMS, Gut, and Water. Relative abundances across communities in descending taxonomic level; Phyla **(A)**, Class **(B)**, Order **(C)**, and Genus **(D)**. Taxonomic groups with abundances less than 0.001% are contained within “Other”.

To broadly describe differences between the communities seen in the beta-diversity metrics, we examined the phyla within each community. We found that the gut community was dramatically different from the other samples, dominated by Fusobacteria (40.3%), Firmicutes (21.4%), Proteobacteria (15.5%), and Bacteroidetes (13.6%) (Figure 2A). The water was dominated by a high level of Proteobacteria (42.7%), and Actinobacteria (35.1%) followed by lower levels of Bacteroidetes (7.8%) and Verrucomicrobia (7.76%). An important caveat is that since the water microbiome was collected in one small time frame, it is likely that the levels of Verrucomicrobia, Actinobacteria, and other taxa could change dramatically with the season. The same can also be said for the other communities.

The SMS microbiota was also predominantly made up of Proteobacteria (56.7%), in this case followed by a smaller abundance of Bacteroidetes (9.8%) and Actinobacteria (8.5%). LEfSe analysis revealed that despite the continuous exposure of the SMS to water containing Verrucomicrobia and Actinobacteria, it contained significantly lower levels of these phyla (LDA > 4; *p*-values < 0.001), while containing significantly more Proteobacteria, Acidobacteria, Planctomycetes, and Cyanobacteria (LDA > 4; *p*-values < 0.01; Supplementary Figures S2A,B). These results begin to reveal that both the SMS and the gut support unique microbial communities, despite persistent exposure to the water environment.

We also looked at the communities at the individual fish level and found that although there was some variability in the number of unique ASVs (Supplementary Table S2), the over-all inter-individual taxonomic composition was fairly consistent across samples, with the exception of fish 4 (Supplementary Figures S1A,B). This fish was dominated by the phylum Spirochaetes, classified further into *Brevinema* (genus) of the Brevinemataceae family (Supplementary Figures S1A,B). This genus has been hypothesized to be a potential opportunistic pathogen of Atlantic salmon (Brown et al., 2019). In that same fish, we found that the SMS contained more *Dependentiae* (genus) and a taxa characterized as “metagenome” from the class Babeliales. Both of these taxa are possible protist endosymbionts (Pagnier et al., 2015; Yeoh et al., 2016; Deeg et al., 2019).

### Microbial Composition – Families

**FIGURE 3:**
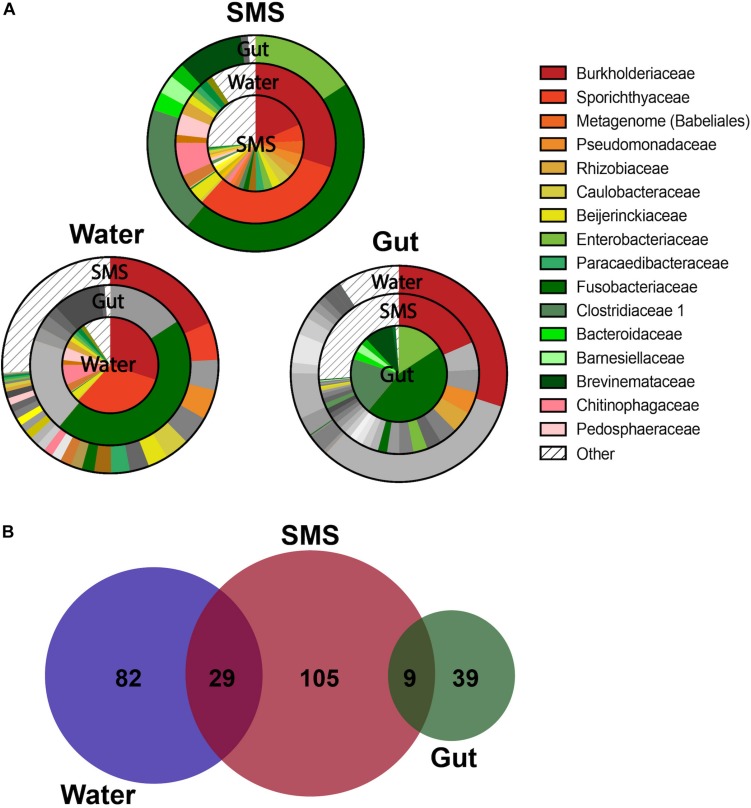
Overlapping Taxonomy and Uniqueness Between SMS, Gut, and Water Communities. **(A)** Overlapping charts highlight the shared families between the communities, with the centers serving as the focus of the surrounding pie charts. For the surrounding communities, if a group is not present within the center community at a greater than 0.001% abundance, than it is grayed out. If the family is present, then it is colored at the appropriate proportion to indicate its relative abundance. Specific families were grouped into “Other” if they constituted less than 0.001% of their own microbiome. For differences in family abundances, refer to Figure 4A and Supplementary Figure S3. **(B)** ASVs are depicted in a proportional Venn Diagram with a 0.01% abundance cutoff.

As we described our communities at lower taxonomic levels (class, order, genus), we found that the differences were more than skin deep. At more specific taxonomic identifications, the divergence between the bacteria living in the SMS, the gut, and the surrounding waters became more apparent (Figures 2A–D). However, the most interesting differences were found at the family level, highlighted by visualizing shared bacterial taxonomy from different sample types using overlapping pie charts (Figure 3). The center of each chart serves as the focus, and then each surrounding ring highlights only those families that are present in the center pie at greater than 0.001%. We are, in this manner, able to color in the similarities and distinctly gray out the dissimilarities between the distinct communities.

**FIGURE 4:**
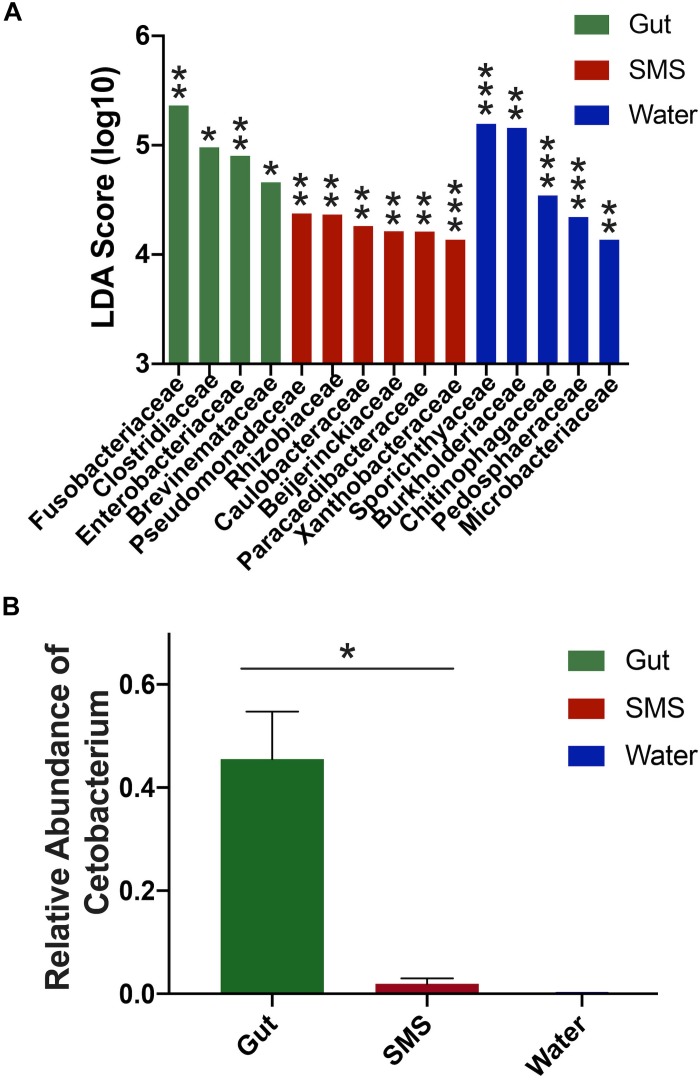
Differences in abundances between SMS, Gut, and Water Communities. **(A)** LDA scores were calculated using LEfSe and indicate families associated with their respective community. Significance cutoff > 4.0 LDA score (log10). For full list of LDA and *p*-values, refer to Supplementary Data S1; ^∗^*p* < 0.05, ^∗∗^*p* < 0.01, ^∗∗∗^*p* < 0.001. **(B)** The gut microbiome exhibited a higher proportion of *Cetobacterium* compared to its SMS and water counterparts, (Mann–Whitney, *p*-value = 0.031).

First, we focused on the SMS community (Figure 3A-SMS), which had the highest alpha diversity (Figures 1A,B). While families found in the SMS makeup 98.9% of the gut microbiome, they tend to differ dramatically in abundance (ex. Fusobacteriaceae, Enterobacteriaceae, Clostridiaceae). For example, Fusobacteriaceae is present in both communities, but comprises 45.3% of the gut community compared to 0.018% found in the SMS (*p*-value < 0.01, Figure 4A). There was a high degree of overlap between the SMS and water communities, with nearly all families (99.4%) found in the SMS also found in the water. Here it is important to reiterate our earlier point that the SMS microbiome is constantly exposed to water, and to add to that, when wet fish skin is sampled, the surrounding water is inadvertently sampled as well. However, as with the gut, the abundances of many families were highly divergent, suggesting that the SMS can establish a unique community despite the constant water contact. Several families, all from the phylum Proteobacteria, were enriched (LDA score > 4.0; *p*-values < 0.01) within the SMS community compared to both of the other communities, including Pseudomonadaceae, Rhizobiaceae, Caulobacteraceae, Beijerinckiaceae, Paracaedibacteraceae, and Xanthobacteraceae (Figure 4A; for all significantly associated families refer to Supplementary Figure S3 and Supplementary Data S1).

We then put the water community at the center of our analysis (Figure 3A-Water). As seen in the previous comparison, there was a high degree of overlap between the families in the water and SMS communities, with the families found in the water comprising 63.6% of the SMS community. On the other hand, we found that the gut contained mainly one family from the water: Fusobacteriaceae. As noted previously, this family was present at 45.3% abundance in the gut but comprises only 0.0001% of the water samples. Families significantly enriched within the water compared to the other communities (LDA score > 4.0; *p*-values < 0.01) were Sporichthyaceae, Burkholderiaceae, Chitinophagaceae, Pedosphaeraceae, and Microbacteriaceae (Figure 4A; for all significantly associated families refer to Supplementary Figure S3 and Supplementary Data S1). Contrary to the Proteobacteria-specific enrichment in the SMS, the families enriched in the water belong to several phyla: Actinobacteria, Proteobacteria, Bacteroidetes, and Verrucomicrobia. These data indicate that the water has more overlap with the SMS than the gut.

Putting the gut community at the center (Figure 3A-Gut) further demonstrates how distinct the taxonomy of this community is from the SMS and the water. The results show that the gut is highly divergent from the SMS, highlighted by the fact that the families found in the gut make up a low proportion of the SMS microbiota and compounded by the low number of overlapping families. The families found in the gut comprised 34.8% of the SMS, which was unsurprising due to the low diversity of the gut samples relative to the SMS. As mentioned before, the gut and water communities were even more different, sharing mainly the families Fusobacteriaceae and Burkholderiaceae at vastly different abundances. In addition to Fusobacteriaceae; Clostridiaceae, Enterobacteriaceae and Brevinemataceae were enriched (LDA scores > 4.0; *p*-values < 0.05) within the gut community (Figure 4A; for all significantly associated families refer to Supplementary Figure S3 and Supplementary Data S1). Given the dominance of Fusobacteriaceae in the gut, we examined the composition of the family and found that it was primarily composed of the genus *Cetobacterium* (Supplementary Data S2). We specifically compared the abundance of *Cetobacterium* in the gut, SMS, and water, finding that it made up a higher proportion of the gut (45.5 ± 9.2%) than the skin mucosa (1.93 ± 1.08%) or the water (0.015 ± 0.05%) (Figure 4B; *p*-values = 0.031 and 0.250). Overall, the gut community is both the least diverse and the families present in the gut are found at low abundances or not at all in the SMS and water communities.

### Microbial Composition – ASVs

To further highlight the divergence between the microbiota, we examined differences in composition at the ASV level. This analysis resulted in further separation between the gut and water communities (Figure 3B). At a threshold of > 0.01% abundance there was no overlap between the gut and water communities, while the SMS still shared some ASVs with the gut (9 ASVs) and the water (29 ASVs). The number of unique ASVs in each community echoed the alpha-diversity findings, with 105 unique ASVs in the SMS, 82 in the water, and 39 in the gut. These results reinforce the uniqueness of each community, as well as the high diversity of the SMS and the water and the relatively low diversity of the gut community. This may be consistent with the constant exposure of the skin mucosa to bacteria in the water; bacteria detected in SMS samples likely include taxa seeded from the surrounding water and driven by similar environmental factors such as salinity, stress, and pH. These results are consistent with the pattern found using the Bray-Curtis and weighted Unifrac indices of beta-diversity (Figures 1C,D). Furthermore, this data supports the establishment and persistence of different microbial communities at different sites on the fish, distinct from both each other and from the microbes living in the surrounding environment.

### Predictive Function of the Microbiomes

To predict the functional differences between the SMS, gut, and water communities we used PICRUSt2 (Douglas et al., 2019). This program uses the 16S content of a community to infer the metagenomic content, and then uses this information to predict the abundances of gene families and pathways based on a number of databases. While this pipeline does not directly measure gene content, it allows us to develop hypotheses about the functional capacity of the taxa in each community. We analyzed the MetaCyc pathways that were specifically associated with each of three communities. Compared to the SMS and the water microbiota, simple and complex carbon metabolism as well as nucleotide biosynthesis pathways were enriched within the gut microbiome (Supplementary Figures S4, S5C and Supplementary Data S3). Additionally, the gut was enriched for B-vitamin biosynthesis pathways, including vitamin B12. Lastly, cell wall and envelope biosynthesis pathways were enriched in the gut, including phospholipid biosynthesis, LPS and S-layer biosynthesis, and peptidoglycan biosynthesis (Supplementary Figures S4, S5C and Supplementary Data S3). In terms of the SMS community, we found an enrichment of antibiotic biosynthesis, photosynthesis, and aromatic compound degradation pathways (Supplementary Figures S4, S5B and Supplementary Data S3). Also, within the SMS, we identified an enrichment of ubiquinone biosynthesis, which has been associated with aerobic Gram-negative bacteria (Collins and Jones, 1981; Meganathan and Kwon, 2009). Conversely, menaquinone biosynthesis pathways associated with aerobic Gram-positive bacteria or anaerobic bacteria in general (Collins and Jones, 1981; Meganathan and Kwon, 2009), were enriched in the water microbiome (Supplementary Figures S4, S5A and Supplementary Data S3). We also found an enrichment in lignin-associated aromatic compound degradation pathways in the water, which is unsurprising as lignin is a common polymer found in the water (Benner et al., 1986; Hernes and Benner, 2003; Osburn et al., 2016; Santos et al., 2019).

## Discussion

This study characterized and compared the SMS and gut microbial communities of the northern pike and their surrounding environment, and found that each community harbored a unique microbial profile despite frequent exposure to microbiota in the surrounding water. In terms of diversity (Figures 1A,B), the gut harbored a lower alpha-diversity compared to the SMS and the water. Other freshwater fish, including the rainbow trout (*Oncorhynchus mykiss*) and the tambaqui (*Colossoma macropomum*), display a similar trend, with a lower alpha diversity in the gut than the SMS (Lowrey et al., 2015; Sylvain et al., 2016). Together, these results suggest that the mucosal surface of piscine skin can support a richer and more diverse community than the gut. Interestingly, the carnivorous diet of the northern pike may influence its gut microbiome diversity. A change in macronutrient intake can rapidly alter the human gut microbiome (David et al., 2014), a trend that has also been reflected in rainbow trout (Desai et al., 2012). In fact, fish feeding habits are a major determinant of GI tract diversity, and several studies have indicated that carnivorous fish have lower gut microbiota diversity that omnivores or herbivores (Larsen et al., 2014; Wang et al., 2018; Butt and Volkoff, 2019). Strikingly, a study by He et al. (2013) showed that even given the same feed of crude protein, fat, and crude fiber and in the same rearing environment, different species of freshwater carp exhibited different levels of bacterial species depending on their trophic level; specifically, diversity decreased from omnivorous to herbivorous to carnivorous. Thus, it is possible that the carnivorous diet of the northern pike influences the low diversity seen in the gut microbiome.

Similarly, taxonomic analysis of the northern pike GI tract indicates that this gut environment harbors a microbial community consistent with that of other freshwater carnivorous species. Specifically, the gut was dominated largely by Fusobacteria (Figure 2A), further classified to the family Fusobacteriaceae and the genus *Cetobacterium*; this lineage comprised more than 40% of the pike gut community. This anaerobic genus (Tsuchiya et al., 2007) has been found in a variety of freshwater fish guts, commonly constituting over 70% of 16S amplicon sequences (Larsen et al., 2014; Tarnecki et al., 2017). Other omnivorous or carnivorous species also harbor *Cetobacterium*, including a number of carp species (Prussian, grass, silver, bighead, common, and crucian carp) as well as rainbow trout, Nile tilapia, Chinese perch, channel catfish, largemouth bass, and bluegill (van Kessel et al., 2011; Larsen et al., 2014; Ye et al., 2014; Etyemez and Balcazar, 2015; Giatsis et al., 2015; Li et al., 2015; Eichmiller et al., 2016; Yan et al., 2016; Zhang et al., 2016; Lyons et al., 2017). Interestingly, the largemouth bass, which consumes a similar diet to the pike (Soupir et al., 2000) also shares two genera in large proportion – the aforementioned *Cetobacterium* and a potential human pathogen *Plesiomonas* (Larsen et al., 2014). These two genera have also been found as core members of other piscivorous microbiomes including perch and pike-perch (*Perca fluviatilis* and *Sander lucioperca*) (Kashinskaya et al., 2018). While *Cetobacterium* itself occurs at higher abundancies in the GI tract of carnivorous and omnivorous compared to herbivorous species (Liu et al., 2016), not all freshwater predators harbor *Cetobacterium* (Llewellyn et al., 2016), indicating that there are other factors driving the establishment of this genus, some of which include seasonality (Ray, 2016; Tarnecki et al., 2017), salinity (Schmidt et al., 2015), and B12 availability (Tsuchiya et al., 2007). Together, these studies suggest that the abundance of this genus is linked to trophic level and our results support the idea that *Cetobacterium* may play a significant role in the GI tract of piscivorous fish.

In terms of function, *Cetobacterium* is known to synthesize cobalamin, also known as vitamin B12, and to prevent the growth of pathogens (Sugita et al., 1996; Tsuchiya et al., 2007). Accordingly, we found that the gut community was associated with several pathways for the biosynthesis and salvage of vitamin B12, as well as for the biosynthesis of other B vitamins (Supplementary Figures S4, S5C and Supplementary Data S3). In the SMS community, we noted an association with a number of aromatic compound degradation pathways, including several for toluene degradation. This may be due to the enrichment in this community of the family *Pseudomonadaceae* (Figure 4), which includes several species with the capacity for degradation of these compounds (Zylstra et al., 1988; Otenio et al., 2005; Nogales et al., 2017). On the other hand, the water community was associated with several pathways for the degradation of lignin derivatives such as vanillin and gallates (Supplementary Figures S4, S5A and Supplementary Data S3; de Gonzalo et al., 2016; Kamimura et al., 2017). As lignins are common terrestrially derived organic molecules found in aquatic ecosystems (Benner et al., 1986; Hernes and Benner, 2003; Osburn et al., 2016; Santos et al., 2019), this suggests that the presence of this carbon source in the water may influence the makeup and function of the microbial community. These observations were generated with PICRUSt2, which uses 16S rRNA amplicon sequencing to assign presence or abundance of gene pathways based on the gene content of previously sequenced bacteria. Since many of the bacteria found in piscine microbiomes have not been fully annotated, the assigned genetic function may be skewed toward fully annotated, terrestrial bacteria.

At the phylum level, the gut and SMS contained taxa in different proportions from both each other and the water, withstanding the constant introduction of bacteria from the environment. Consistent with previous freshwater reports (Wang et al., 2018), we found that the gut contained predominantly Fusobacteria, followed by Firmicutes, Proteobacteria, and Bacteroidetes. The microbiota of the SMS, in contrast, was high in Proteobacteria with lower proportions of Bacteroidetes and Actinobacteria. Finally, the water was dominated by Proteobacteria and Actinobacteria, with lower levels of Bacteroides and Verrucomicrobia. While aqueous environments are thought to provide a crucial avenue for bacterial colonization (Ingerslev et al., 2014; Galbraith et al., 2018), these differences even at a high taxonomic level indicate the establishment of a microbiome with specificity to the SMS and GI tract despite the constant influx of water.

At lower taxonomic levels, the composition of the SMS and the gut exhibited increasingly divergent bacterial profiles, which were also distinct from that of the surrounding water (Figures 2B–D). Most of the families within the SMS community overlapped with the gut and water communities (Figure 3A-SMS), but they were often at vastly different proportions (for example, Fusobacteriaceae). Many families, all derived from the phylum Proteobacteria, were specifically enriched in the SMS compared to the gut and water (Figure 4A; ex. Pseudomonadaceae, Rhizobiaceae, Caulobacteraceae, Beijerinckiaceae, Paracaedibacteraceae, and Xanthobacteraceae). Pseudomonadaceae, further classified down into *Pseudomonas*, is a common member of SMS communities. This genus has been found in both freshwater and saltwater fish including the channel catfish, brook trout, red snapper, striped mullet, sand seatrout, pinfish, and spotted seatrout (Larsen et al., 2013; Mohammed and Arias, 2015; Galbraith et al., 2018). Interestingly, several Proteobacteria – *Acinetobacter, Polynucleobacter, and Methylobacterium* – that were present in over 85% of our samples have previously been found in SMS of other fish alongside with *Pseudomonas*. The SMS of gibel carp, black bream, striped mullet, red snapper, and pinfish exhibit both *Acinetobacter* and *Pseudomonas* in conjunction (Wang et al., 2010; Larsen et al., 2013). *Methylobacterium* have been documented to produce poly-b-hydroxybutyrates, which can inhibit the growth of potential pathogens (Defoirdt et al., 2007; Halet et al., 2007). The brook trout, whose habitat ranges overlap with the northern pike, shares all four of these SMS inhabitants (Boutin et al., 2013; Galbraith et al., 2018), suggesting a shared influence of environmental factors such as salinity (Lokesh and Kiron, 2016; Carda-Dieguez et al., 2017), sediment (Hess et al., 2015), stress (Boutin et al., 2013), and pH (Sylvain et al., 2016) on the establishment of the SMS community.

The gut was also strongly associated with a variety of families (Figure 4 and Supplementary Figure S3; ex. Fusobacteriaceae, Clostridaceae, Enterobacteriaceae, Brevinemataceae), demonstrating a divergence of the gut from the SMS and the surrounding water. In addition, several families were enriched in the water compared to the SMS and gut, including Sporichthyaceae, Burkholderiaceae, Chitinophagaceae, and Pedosphaeraceae (Figure 4A). Overall, our results suggest the establishment of communities specific to the gut, SMS, and the water. We found the SMS and the water samples clustered separately using the Bray-Curtis index (based on ASV abundance), but were not distinguishable using the Weighted UniFrac metric (incorporating both phylogeny and ASV abundance); thus, the separation identified by the Bray–Curtis may arise from closely related ASVs. The gut clustered separately from the water and SMS for both metrics. The distinction between the communities is further supported at the ASV level, with 73.4, 81.3, and 73.8% of the ASVs only found in the SMS, gut, and water, respectively (Figure 3B). In fact, we found that no ASV overlapped between the gut and the water, in contrast to a study which found that 29.45% of OTUs in the gut microbiota of turbots were shared with the water (Xing et al., 2013). However, this study used a similarity cutoff of 97% identity, while our study had a 99% cutoff, as well denoising strategies to obtain ASVs and a 0.01% abundance restriction. This result at the ASV level is perhaps the strongest indication that each body site harbors a distinct microbiome. Overall, this work supports the idea that while fish are constantly exposed to the microbes of their aqueous habitat, their niches represent unique environments and are able to establish communities that are highly divergent at multiple scales.

This study has several limitations intrinsic to methodology and sample size that must be acknowledged. First of all, accurate ASV annotation requires robust 16S databases that include organisms from diverse environments. However, since freshwater fish microbiomes are not as well-studied as the murine or human microbiome, it is likely that many of the unique 16S sequences that are found in these communities are not yet included in the Silva database. Second, we must acknowledge limitations arising from sample size. While our data clearly shows interesting and statistically significant differences in community structure between the water, the SMS, and the gut, it is also possible that a larger sample size could detect more differences with higher statistical certainty. Overall, we are heartened that the inter-individual variability of the taxonomic composition of each sample each site was relatively low indicating that a relatively small sample size could provide a reliable description of each community. Finally, because our sampling covered a single river during one season, it is possible that changing both of these factors could impact the composition on the microbiota. Future studies could be conducted to define the impacts of location and season on the composition of the *E. lucius* microbiome.

## Data Availability

The datasets generated for this study can be accessed from the Brown Digital Repository (https://repository.library.brown.edu/studio/item/bdr:864309/), where it will be embargoed until publication.

## Ethics Statement

According to the Public Health Service Policy on Humane Care and Use of Laboratory Animals (PHS Policy), this study is exempt because it utilized microbiome samples collected from dead fish that were not killed, collected, or manipulated antemortem for the purpose of this study. All samples were collected postmortem from fish harvested from licensed recreational fishermen who gave permission for us to sample their catch.

## Author Contributions

ER, BK, and PB contributed to the conception and design of the study. BK extracted and processed the samples. ER, AR-N, and BK performed the statistical analysis. ER wrote the first draft of the manuscript. All authors contributed to the manuscript revision and approval of the submitted version.

## Conflict of Interest Statement

The authors declare that the research was conducted in the absence of any commercial or financial relationships that could be construed as a potential conflict of interest.

## Footnotes

1 https://qiime2.org/
