# Supplementary material for: Defining the Distinct Skin and Gut Microbiomes of the Northern Pike (Esox lucius)
Source: Front Microbiol. 2019 Sep 12;10:2118. doi: 10.3389/fmicb.2019.02118 (PMC6751255; doi:10.3389/fmicb.2019.02118)
Supplement: Supplementary file 1 [file Data_Sheet_1.pdf]

Table S1: GPS Coordinates for Sample Collections. Coordinates begin in Lac Cambrai, Quebec, Canada and follow southwest down the Chochocouane River. Fish were measured for total length from the snout to the end of the pinched tail.

Collection Site	Coordinates	Sample	Length
1	47°57'36.2"N 76°39'05.8"W	SMS01<br>Gut01	53 cm
2	47°55'06.9"N 76°46'38.3"W	SMS02<br>Gut02	51 cm
3	47°53'00.1"N 76°46'07.4"W	SMS03<br>Gut03	46 cm
4	47°53'00.1"N 76°46'07.4"W	SMS04<br>Gut04	53 cm
5	47°48'49.6"N 76°51'42.8"W	SMS05<br>Gut05	71 cm
6	47°43'39.9"N 77°02'38.3"W	SMS06<br>Gut06	91 cm
7	47°41'15.4"N 77°04'01.6"W	SMS07<br>Gut07	51 cm
8	47°40'04.7"N 77°04'33.0"W	Gut08	64 cm

Table S2: Individual Raw Read and ASV Counts.

Sample	Number of Raw Read Counts	Unique ASV Counts
SMS01	52,627	823
SMS02	56,017	334
SMS03	43,380	512
SMS04	87,777	498
SMS05	11,049	318
SMS06	122,348	727
SMS07	99,797	337
Gut01	148,005	27
Gut02	99,919	19
Gut03	116,559	30
Gut04	169,618	43
Gut05	159,739	49
Gut06	178,159	48
Gut07	103,163	18
Gut08	94,814	21
Water01	125,612	391
Water02	107,543	385
Water03	34,814	222

Figure S1—Taxonomic Abundances of Individual Samples and Beta Diversity in Relation to Fish Length. Differences in taxonomic relative abundance between individual samples at the Phylum (A) and Family (B) level. Fish length plotted against PCo1 of the weighted Unifrac (C). Bray-Curtis colored by sampling location (D), squares and circles represent SMS and gut respectively.

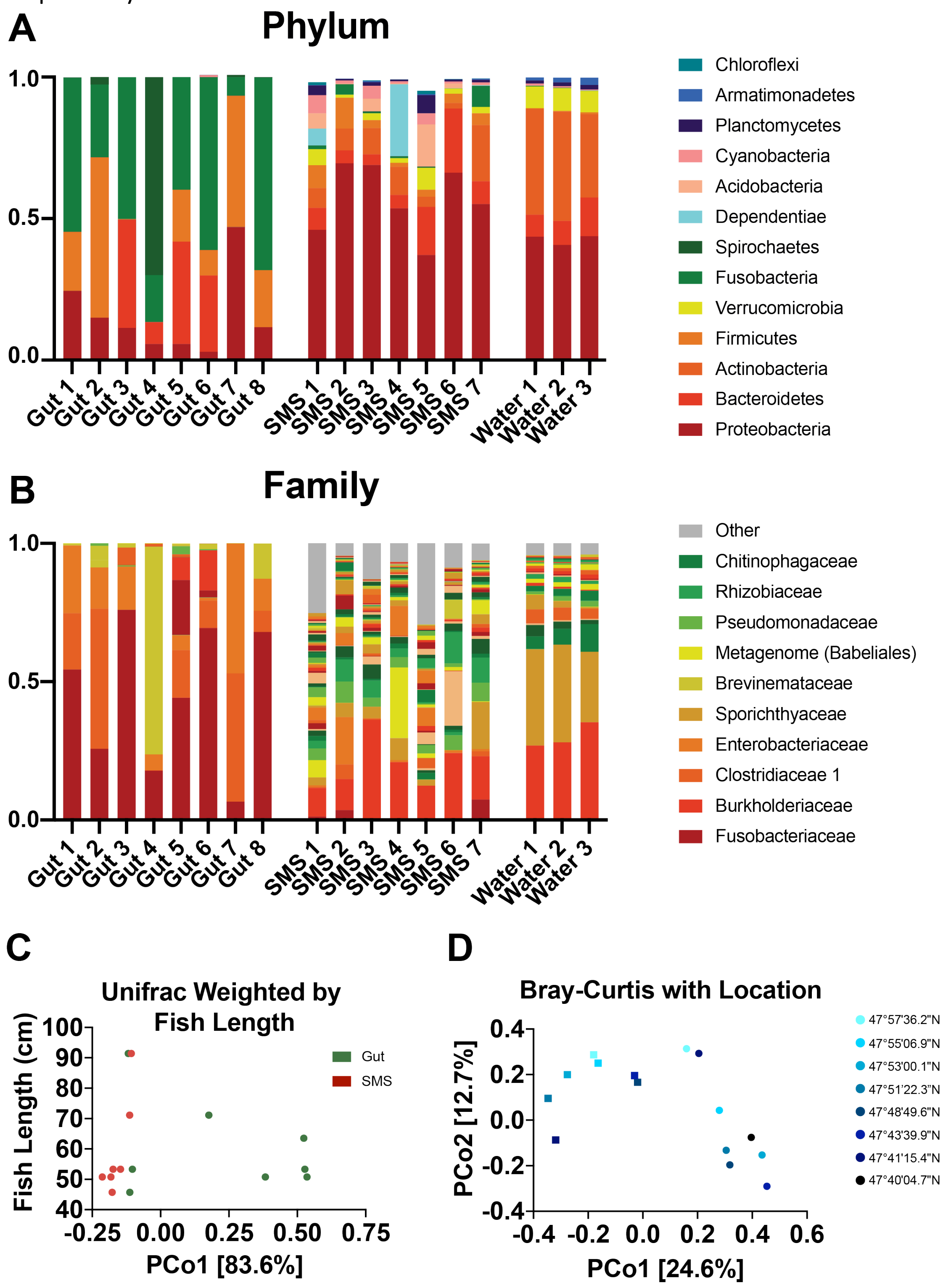

Figure S2: Differences in Phyla Abundances Between Gut, SMS and Water Microbiomes. LDA Scores were calculated using LEfSe and correlate with phyla enriched in their respective biome. Significance cutoffs were (A) > 4.0 and (B) > 2.5 LDA score (log10). For full list of LDA and p-values, refer to Data S3; (*) p< 0.05, (**) p<0.01, (***) p<0.001].

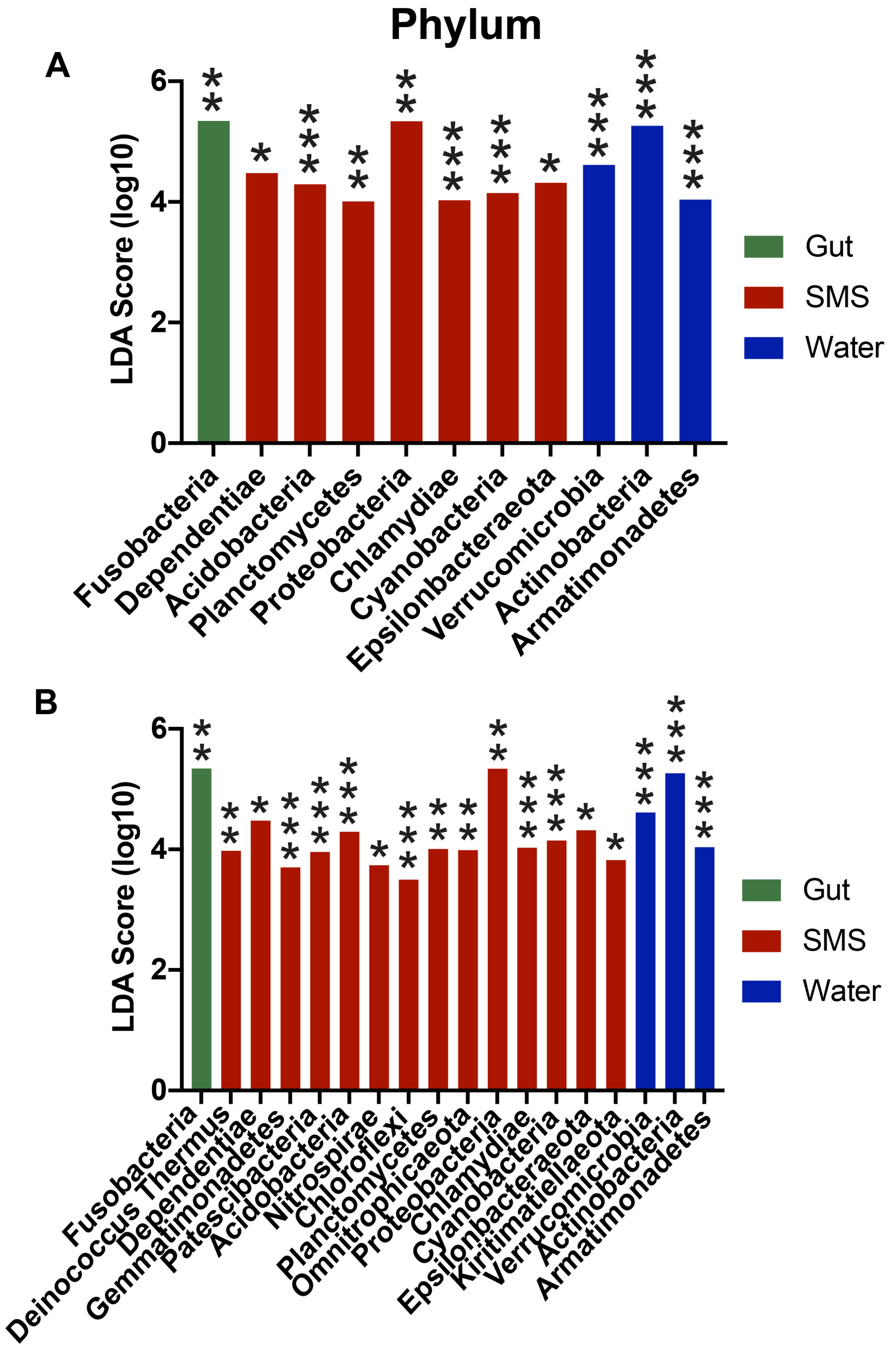

Figure S3: Differences in Family Abundances Between Gut, SMS and Water Microbiomes with cutoff of LDA > 2.5. LDA Scores were calculated using LEfSe and correlate with families enriched in their respective biome. For full list of LDA and corresponding p-values, refer to Data S3.

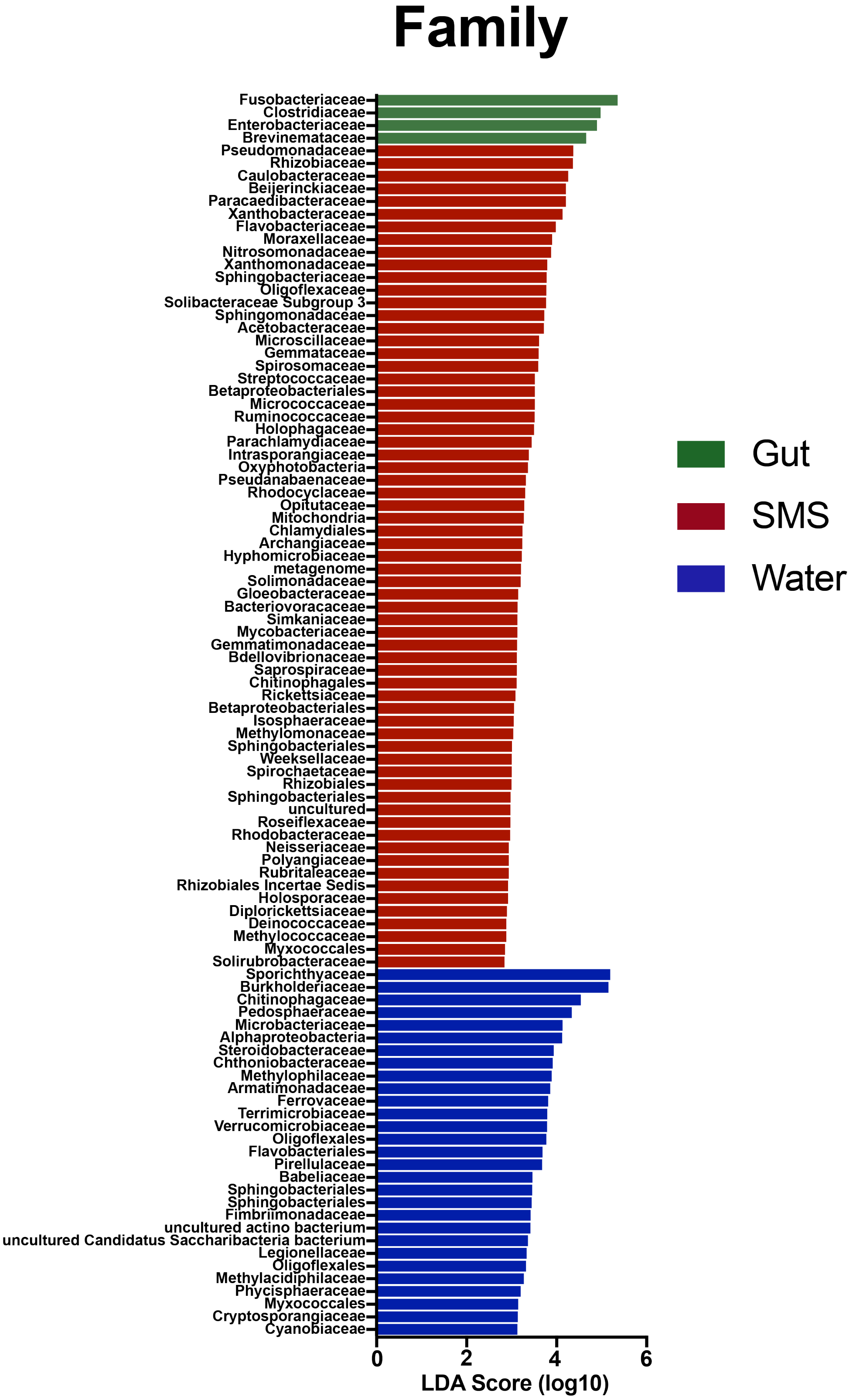

Figure S4: Predicted Metagenomic Function: Summary of Enriched Metacyc Pathways per Gut, SMS and Water Microbiome. Pathways enriched within the microbial sources were predicted via the PICRUSt2 plugin within QIIME2 and mapped into the KEGG Orthologs and Pathways database. Associated pathways were grouped based on general function (for itemized list of pathway refer to Figure S5 and S2 Data).

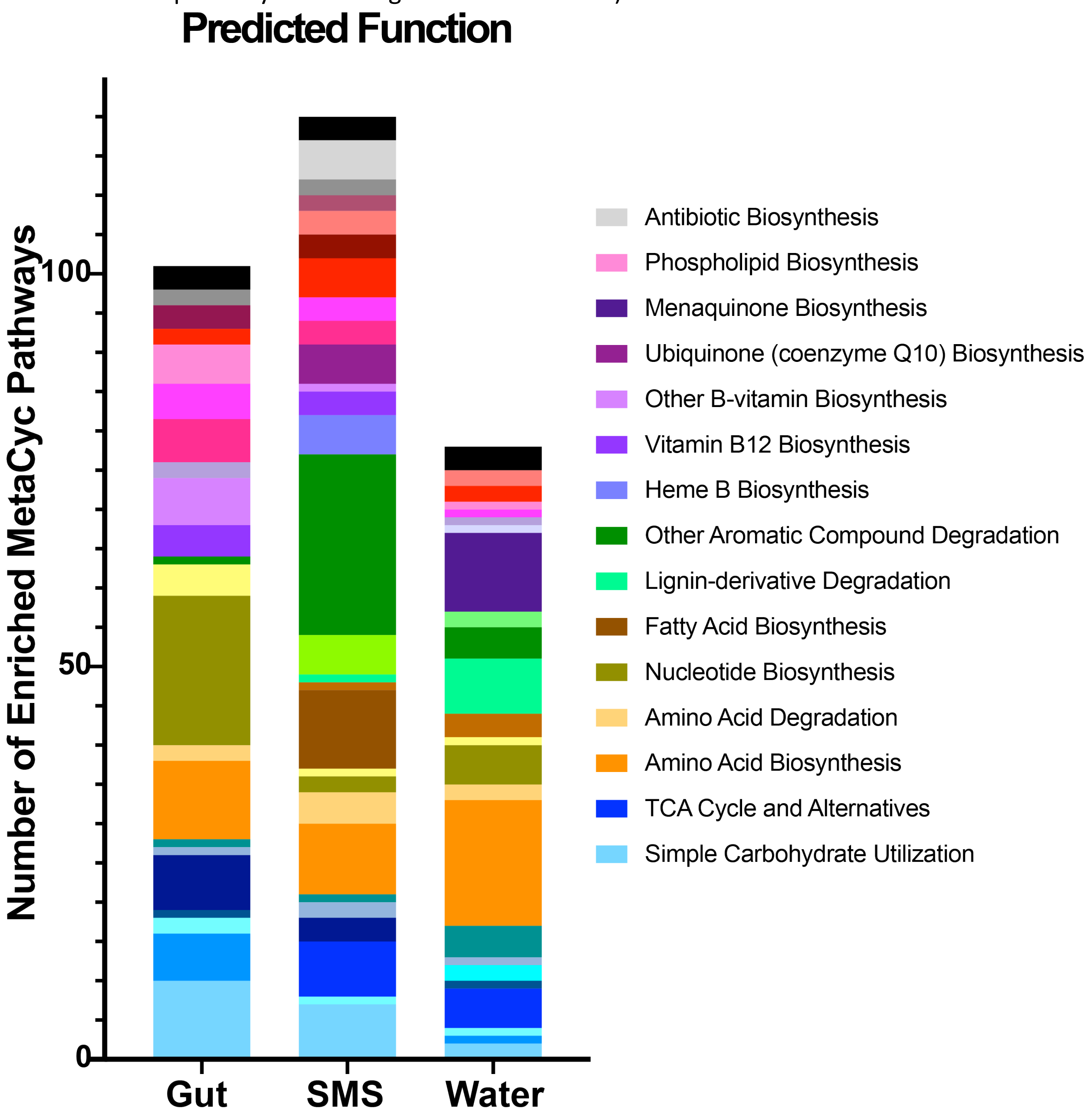

Figure S5: Predicted Metagenomic Function: Metacyc Pathways of the Gut, SMS and Water Microbiomes. Enriched pathways for the water (A), SMS (B), and gut (C) microbiomes. Pathways were predicted via the PICRUSt2 plugin within QIIME2 and mapped into the KEGG Orthologs and Pathways database. Associated pathways were grouped based on general function (for itemized list of pathway refer to S2 Data).

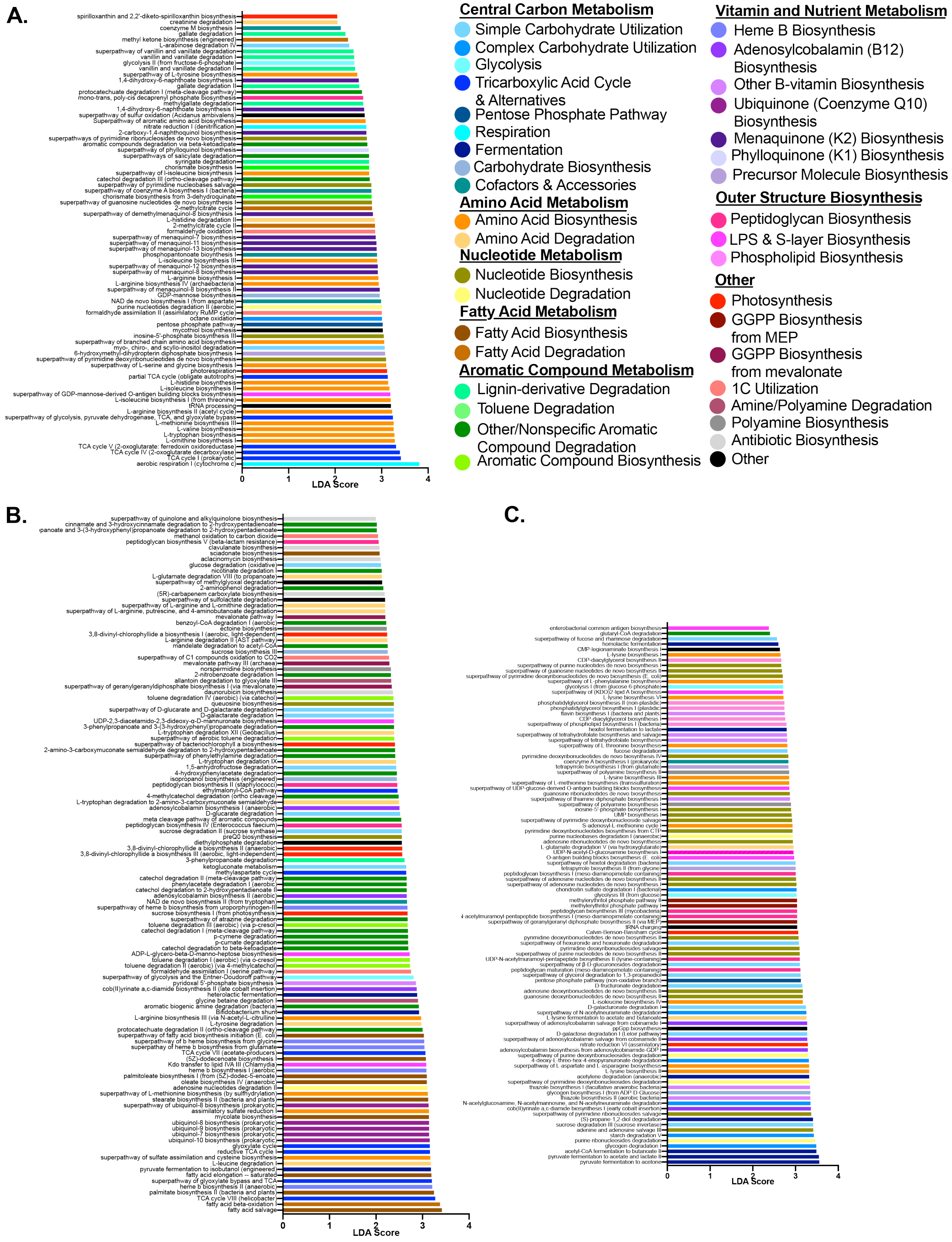

Supplementary Data S1: Differences in Taxonomic Abundances Between Gut, SMS and Water Microbiomes. Each tab includes the significant LDA and p-values calculated by LEfSe; Tab 1 delineates the family-level data and Tab 2 delineates the phyla-level data.

Supplementary Data S2: Per-sample Relative Abundance Data. Each tab includes different taxonomic level data; Tab 1 delineates phylum-level data, Tab 2 delineates class-level data, Tab 3 delineates order-level data, Tab 4 delineates family-level data, Tab 5 delineates genus-level data and Tab 6 delineates ASV-level data**.**

Supplementary Data S3: Predicted Metagenomic Function: Specific Metacyc Pathways Associated with the Gut, SMS, and Water Microbiomes
